# Supplementary material for: Knowledge, attitude and practice towards complementary and alternative medicine and associated factors among health care professionals in public health facilities of Hadiya Zone, South Ethiopia
Source: PLoS One. 2022 Sep 9;17(9):e0274333. doi: 10.1371/journal.pone.0274333 (PMC9462560; doi:10.1371/journal.pone.0274333)
Supplement: S1 Questionnaire — (DOCX) [file pone.0274333.s001.docx]

**S1: Questionnaire**

**Consent form:**

Hello, my name is Belay Erchafo and I am a researcher at Wachemo University College of medicine and Health Sciences, currently am conducting a research to assess Knowledge, Attitude and Practice towards complementary and alternative medicine among health care providers of Hadiya Zone. Thus, I am requesting your cooperation to fill out the survey question which will take about 20 minutes to complete. Participation in this survey will be voluntary, and if you don’t want to participate or if there is any question you don’t want to answer you can skip to the next, or if you choose not to participate you could withdraw at any time. I assure all information that you provide will remain strictly private, and confidentiality of responses would be maintained during and after data collection. Only numbers will be assigned to each copy and no name will be required on the questionnaire. The numbers would facilitate data entry and analysis, so no one can link your identity with the registration numbers. Findings from this research are believed to serve the health system planners to design evidence based programs on integrative medicine. I hope you will participate in the survey as your feedbacks are important. Thank you for your willingness to be my study participant and taking time to fill study questionnaire.

If you have any questions & concerns about the study you should contact:

Department of Public health, College of Medicine &Health Sciences, Wachemo University

**Name:** Belay Erchafo **Mobile Phone:** +251915109228 **Email:** erchafobelay@gmail.com

Do I have your permission to continue? Yes No

If yes, thank and sign on the space provided. Signature------------------

If no, thank and go to the next participant

| **General Information** | Date _____________  Respondent ID ______________  Health facility/Health institution name _______________ |
| --- | --- |

Questioners for the study

Questioners to determine knowledge, attitude, and practice towards complementary and alternative medicine among health care providers in primary health care unit Hadiya Zone, Southern Ethiopia,2020.

| ` **Section 1: Questions to assess Socio Demographic Information** | | |
| --- | --- | --- |
| 001 | How old are you? | Age in years--------------- |
| 002 | What is your sex? | 1.male 2.female |
| 003 | Marital Status | 1.Single 2.Married  3.Divorsed 4.widowed |
| c004 | Which ethnic group would you belong? | 1. Hadiya 2.Kambata  3 Amahara. 4. Oromo  5. Gurage 6.others specify…….. |
| 005 | What is your educational level? | 1.Diploma 2.Degree 3.postgraduate degree |
| 006 | What is your field of study? | 1. Nurse 2.Health officer  3. GP 4.Midwifery  5. Pharmacy 6. Others-------- |
| 007 | What is your work experience? | In years___________ |
| 008 | Type of health facility you are currently working | 1.primary hospital  2. Health center |

Complementary medicine is used **in addition to** standard treatments whereas alternative medicine is used **instead of** standard treatments. Complementary and alternative medicine include

1. Medical herbalism

2. Spiritual/faith healing

3. Traditional bone setting

4. Massage

5. Vitamins and other nutritional therapies

| **Section 2: Questions to assess Knowledge of the Respondent** | | | |
| --- | --- | --- | --- |
| No | Questions | Answers | |
| 201 | Have you ever heard about alternative and complementary medicine? | 1. Yes 2.No | |
| 202 | If yes Q 201 Source of information about complementary and alternative medicine (CAM) | 1. Families, friends, and relatives 2. Health care providers 3. Media (internet, television, radio, and book) 4. Patients using CAM 5. Traditional healers 6. Others | |
| 203 | If yes for Q 201 Which treatments option do you know from complementary and alternative medicine | 1. Medical herbalism 2. Spiritual/faith healing 3. Traditional bone setting 4. Massage 5. Vitamins and other nutritional therapies 6. Others ----- | |
| 204 | Practicing with knowledge of complementary and alternative medicine and modern medicine is superior to practicing with only knowledge of modern medicine | 1. Yes 2. No | |
| 205 | Have you taken pre service training on complementary and alternative medicine? | 1.Yes 2.No 3.I don’t know | |
| 206 | If yes, where do you get? | 1.from University or college  2.from health institution  3.others______________ | |
| 207 | If yes for Q 205, is there clinical attachment? | 1.Yes 2.No 3.I don’t know | |
| 208 | Is health education about risks and benefits of alternative and complementary medicines important? | 1.Yes 2.No | |
| 209 | Do you know harmful effects of complementary and alternative medicine? | 1.Yes 2.No | |
| 210 | If yes for Q 209 Which one is harmful effect of complementary and alternative medicine? (more than one answer is possible) | 1. Diarrhea 2. Vomiting 3. Abdominal pain 4. Skin discoloration 5. Other------- | |
| **Section 3: Questions to assess Attitude of the Respondent** | | | |
| No | Questions | | Answers |
| 301 | How much do you agree with the provision of both CAM and modern medicine in combination | | 1.Strongly agree  2. Agree  3. Neutral  4.Disagree  5. Strongly disagree |
| 302 | Providing both CAM and modern medicine for patients could increase patient satisfaction | | 1.Strongly agree  2. Agree  3. Neutral  4.Disagree  5. Strongly disagree |
| 303 | complementary and alternative medicine therapies can contribute in curing illness | | 1.Strongly agree  2. Agree  3. Neutral  4.Disagree  5. Strongly disagree |
| 304 | complementary and alternative medicine therapies can promote general health and wellness | | 1.Strongly agree  2. Agree  3. Neutral  4.Disagree  5. Strongly disagree |
| 305 | Medical practitioners should be more educated in the use of complementary and alternative medicine | | 1.Strongly agree  2. Agree  3. Neutral  4.Disagree  5. Strongly disagree |
| 306 | How much do you support the incorporation of complementary and alternative medicine in the medical curriculum | | 1.Strongly agree  2. Agree  3. Neutral  4.Disagree  5. Strongly disagree |
| 307 | Would you support complementary and alternative medicine being introduced in a drug formulary | | 1.Strongly agree  2. Agree  3. Neutral  4.Disagree  5. Strongly disagree |
| 308 | Research on the efficacy and safety of complementary and alternative medicine should be performed | | 1.Strongly agree  2. Agree  3. Neutral  4.Disagree  5. Strongly disagree |
| 309 | Provision of wellness centers using complementary and alternative medicine and modern medicine would benefit patients | | 1.Strongly agree  2. Agree  3. Neutral  4.Disagree  5. Strongly disagree |
| 310 | Do you have planned to use alternative and complementary medicine in the future? | | 1.Strongly agree  2. Agree  3. Neutral  4.Disagree  5. Strongly disagree |

| **Section 4: Questions to assess Practice of the Respondent** | | | | |
| --- | --- | --- | --- | --- |
| No | Questions | | Answers | |
| 401 | Do you ask patients about complementary and alternative medicine usage? | | 1.Yes 2.No | |
| 402 | Do you recommend the use of complementary and alternative medicine for your patients? | | 1.Yes 2.No | |
| 403 | Types of CAM commonly recommended for your patients | | 1. Medical herbalism 2. Spiritual/faith healing 3. Traditional bone setting 4. Massage 5. Vitamins and other nutritional therapies 6. Others -----) | |
| 404 | Reasons to Prefer complementary and alternative medicine to modern medicine | | 1. Affordability 2. Accessibility 3. Acceptability 4. Effectiveness | |
| 405 | Have you taken any supplements/herbals in the last 2 years for yourself? | | 1. Yes 2.No | |
| 406 | What other complementary medicine modalities have you used besides supplements /herbals(select that apply) | | 1. Medical herbalism 2. Spiritual/faith healing 3. Traditional bone setting 4. Massage 5. Vitamins and other nutritional therapies 6. Others -----) | |
| 407 | How often do you use supplements or complementary modalities? | | 1. Daily 2. At least once a week 3. At least once a month 4. At least once a year 5. At least once in the past five years 6. Rarely | |
| **Section 5:Health facility related information** | | | | |
|  | | Questions | | Answers |
| 501 | | Do you have facility to deliver complementary and alternative medicine service in your health center? | | 1. Yes 2.No |
| 502 | | Have you taken in- service training on complementary and alternative medicine? | | 1.Yes 2.No |
| 503 | | Do you have experience of regular contact with patients who seek complementary and alternative medicine | | 1.Yes 2.No |

***Thank you for your cooperation to take part in the study!!!!***
