# Supplementary material for: Knowledge, attitude and practice towards complementary and alternative medicine and associated factors among health care professionals in public health facilities of Hadiya Zone, South Ethiopia
Source: PLoS One. 2022 Sep 9;17(9):e0274333. doi: 10.1371/journal.pone.0274333 (PMC9462560; doi:10.1371/journal.pone.0274333)
Supplement: Attachment — Submitted filename: PLOSONE-Reviewer Report.docx [file pone.0274333.s002.docx]

**February 15, 2022**

**Reviewer Report:**

**Comments to the Author**

(1) Brief information about data collection tools and measurement can be given (page ii, line 26).

(2) Information about the reasons for using TAT can be given (page, 1 line 45).

(3) It would be helpful to use a source containing a recent research done on page 2, line 67-69 (Sarman, A., & Uzuntarla, Y. (2022). Attitudes of healthcare workers towards complementary and alternative medicine practices: A cross-sectional study in Turkey. European Journal of Integrative Medicine, 49, 102096. <https://doi.org/https://doi.org/10.1016/j.eujim.2021.102096>. Source address: <https://www.sciencedirect.com/science/article/abs/pii/S1876382021008143>).

(4) Is there any particular reason for not including critically ill healthcare workers? Since those with such diseases tend to use CAM more, it can be written in detail in the limitation section of the study (page 3, line 98-99).

(5) The methodology of the research is stated clearly and comprehensibly. The population and sample selection is appropriate. I think there is no problem with the randomization method (page 3-4-5, line 84-156).

(6) The sum of the column percentages of the marital status of the study participants should be checked. It is stated in the table as 100.1% (page 7, line 167; Table 1)

(7) It would be helpful to use a source containing a recent research done on page 14, line 231-232 (Sarman, A., & Uzuntarla, Y. (2022). Attitudes of healthcare workers towards complementary and alternative medicine practices: A cross-sectional study in Turkey. European Journal of Integrative Medicine, 49, 102096. <https://doi.org/https://doi.org/10.1016/j.eujim.2021.102096>. Source address: <https://www.sciencedirect.com/science/article/abs/pii/S1876382021008143>).

(8) The manuscript is presented in an intelligible fashion and written in standard English.

(9) I cannot advise on statistical analysis of the paper.

**Reviewer Confidential Comments to Editor**

(1) I think the article contains successful results.

(2) A study containing very important and striking data. Therefore, I thank the authors. There are no shortcomings, except for a few minor suggestions. I think it will contribute to the literature if it is published. As I always say, appreciation and decision are yours.

Sincerely.
