# Supplementary material for: Knowledge, attitude and practice towards complementary and alternative medicine and associated factors among health care professionals in public health facilities of Hadiya Zone, South Ethiopia
Source: PLoS One. 2022 Sep 9;17(9):e0274333. doi: 10.1371/journal.pone.0274333 (PMC9462560; doi:10.1371/journal.pone.0274333)
Supplement: Attachment — Submitted filename: Rebuttal letter.docx [file pone.0274333.s003.docx]

PONE-D-21-34689

Knowledge, Attitude and practice towards complementary and alternative medicine among health care professionals in public health facilities of Hadiya zone, South Ethiopia

PLOS ONE

Dear Dr. lubago,

Thank you for submitting your manuscript to PLOS ONE. After careful consideration, we feel that it has merit but does not fully meet PLOS ONE’s publication criteria as it currently stands. Therefore, we invite you to submit a revised version of the manuscript that addresses the points raised during the review process.

Please submit your revised manuscript by Apr 21 2022 11:59PM. If you will need more time than this to complete your revisions, please reply to this message or contact the journal office at [plosone@plos.org](mailto:plosone@plos.org). When you're ready to submit your revision, log on to <https://www.editorialmanager.com/pone/> and select the 'Submissions Needing Revision' folder to locate your manuscript file.

Please include the following items when submitting your revised manuscript:

- A rebuttal letter that responds to each point raised by the academic editor and reviewer(s). You should upload this letter as a separate file labeled 'Response to Reviewers'.
- A marked-up copy of your manuscript that highlights changes made to the original version. You should upload this as a separate file labeled 'Revised Manuscript with Track Changes'.
- An unmarked version of your revised paper without tracked changes. You should upload this as a separate file labeled 'Manuscript'.

If you would like to make changes to your financial disclosure, please include your updated statement in your cover letter. Guidelines for resubmitting your figure files are available below the reviewer comments at the end of this letter.

If applicable, we recommend that you deposit your laboratory protocols in [protocols.io](http://protocols.io) to enhance the reproducibility of your results. Protocols.io assigns your protocol its own identifier (DOI) so that it can be cited independently in the future. For instructions see: <https://journals.plos.org/plosone/s/submission-guidelines#loc-laboratory-protocols>. Additionally, PLOS ONE offers an option for publishing peer-reviewed Lab Protocol articles, which describe protocols hosted on [protocols.io](http://protocols.io). Read more information on sharing protocols at <https://plos.org/protocols?utm_medium=editorial-email&utm_source=authorletters&utm_campaign=protocols>.

We look forward to receiving your revised manuscript.

Kind regards,

Jenny Wilkinson, PhD
Academic Editor
PLOS ONE

Dear Academic Editor and Reviewers:

Thank you for providing us an opportunity to revise and resubmit our paper. Below we address all the questions and concerns raised by the academic editor and reviewers in a point-by-point fashion.

Journal Requirements:

When submitting your revision, we need you to address these additional requirements.

1. Please ensure that your manuscript meets PLOS ONE's style requirements, including those for file naming. The PLOS ONE style templates can be found at
   <https://journals.plos.org/plosone/s/file?id=wjVg/PLOSOne_formatting_sample_main_body.pdf> and 
   <https://journals.plos.org/plosone/s/file?id=ba62/PLOSOne_formatting_sample_title_authors_affiliations.pdf>

Response: the revised manuscript follows the plose one format

2. Please amend your current ethics statement to address the following concerns:
   a) Did participants provide their written or verbal informed consent to participate in this study?

Response: written consent

b) If consent was verbal, please explain i) why written consent was not obtained,

ii) how you documented participant consent,

Response: The participants' anonymity was maintained by instructing them not to write their names; the individual's information was not disclosed to any other person or party; and informed consent was obtained. They were told that they had the full right to participate or refuse participation in the study. They have the right to stop in the meantime while completing questioners if they do not feel comfortable, keeping in mind the rationale of the study and the benefit of their response.

and iii) whether the ethics committees/IRB approved this consent procedure.

Response: The study was conducted after securing ethical approval from the Ethical Review Committee of Wachemo University, College of Medicine and Health Sciences. All the participants were well informed about the purpose of the study, benefits and risks associated with the study, written consent was secured from each study participant before collecting the data. The participants were also informed that their responses would be kept confidential and their names would not be mentioned.

3. Thank you for stating the following in the Acknowledgments Section of your manuscript:

We would like to forward our gratitude to Wachemo University for providing fund for this study.

We note that you have provided additional information within the Acknowledgements Section that is not currently declared in your Funding Statement. Please note that funding information should not appear in the Acknowledgments section or other areas of your manuscript. We will only publish funding information present in the Funding Statement section of the online submission form. 
Please remove any funding-related text from the manuscript and let us know how you would like to update your Funding Statement. Currently, your Funding Statement reads as follows:

The author(s) received no specific funding for this work.

Response: we arranged accordingly

Please include your amended statements within your cover letter; we will change the online submission form on your behalf.

Response: Regarding financial disclosure this study was funded by Wachemo University through a staff research grant. The funders had no role in study design, data collection and analysis, the decision to publish, or the preparation of the manuscript.

3. PLOS requires an ORCID iD for the corresponding author in Editorial Manager on papers submitted after December 6th, 2016. Please ensure that you have an ORCID iD and that it is validated in Editorial Manager. To do this, go to ‘Update my Information’ (in the upper left-hand corner of the main menu), and click on the Fetch/Validate link next to the ORCID field. This will take you to the ORCID site and allow you to create a new iD or authenticate a pre-existing iD in Editorial Manager. Please see the following video for instructions on linking an ORCID iD to your Editorial Manager account: <https://www.youtube.com/watch?v=_xcclfuvtxQ>

Response: a pre-existing ORCID ID was authenticated

4. Your ethics statement should only appear in the Methods section of your manuscript. If your ethics statement is written in any section besides the Methods, please move it to the Methods section and delete it from any other section. Please ensure that your ethics statement is included in your manuscript, as the ethics statement entered into the online submission form will not be published alongside your manuscript.

Response: we organized ethical statement in methods part

5. We note you have included a table to which you do not refer in the text of your manuscript. Please ensure that you refer to Table 3, 4 and 5 in your text; if accepted, production will need this reference to link the reader to the Table.

Response: we arranged accordingly

6. Please include captions for your Supporting Information files at the end of your manuscript, and update any in-text citations to match accordingly. Please see our Supporting Information guidelines for more information: <http://journals.plos.org/plosone/s/supporting-information>.

Response: we arranged accordingly

5. Review Comments to the Author

Please use the space provided to explain your answers to the questions above. You may also include additional comments for the author, including concerns about dual publication, research ethics, or publication ethics. (Please upload your review as an attachment if it exceeds 20,000 characters)

Reviewer #1: (1) Brief information about data collection tools and measurement can be given (page ii, line 26).

Response: we appreciate the comment and we have incorporated it in the revised copy. The data were collected using a standardized self-administered and pre-tested questionnaire that was adapted from different literatures.

(2) Information about the reasons for using TAT can be given (page, 1 line 45).

Response: As is obvious, the majority of society in developing nations rely on complementary and alternative medicine for a variety of reasons, including accessibility, price, and a perceived assumption that it is safer and more effective than modern medicine.

(3) It would be helpful to use a source containing a recent research done on page 2, line 67-69 (Sarman, A., & Uzuntarla, Y. (2022). Attitudes of healthcare workers towards complementary and alternative medicine practices: A cross-sectional study in Turkey. European Journal of Integrative Medicine, 49, 102096. [https://doi.org/https://doi.org/10.1016/j.eujim.2021.102096](https://doi.org/https:/doi.org/10.1016/j.eujim.2021.102096). Source address: <https://www.sciencedirect.com/science/article/abs/pii/S1876382021008143>).

Response: we appreciate the comment and we have incorporated it in the revised copy.

(4) Is there any particular reason for not including critically ill healthcare workers? Since those with such diseases tend to use CAM more, it can be written in detail in the limitation section of the study (page 3, line 98-99).

Response: we consider health care professionals who are critically ill are unable to respond for the questioner due to the physiological problem and we incorporated this as the limitation of the study in revised manuscript

(5) The methodology of the research is stated clearly and comprehensibly. The population and sample selection is appropriate. I think there is no problem with the randomization method (page 3-4-5, line 84-d

Response: the population and sample selection were appropriate and randomization were done properly

(6) The sum of the column percentages of the marital status of the study participants should be checked. It is stated in the table as 100.1% (page 7, line 167; Table 1)

Response: we appreciate the comment and we have corrected in revised manuscript

(7) It would be helpful to use a source containing a recent research done on page 14, line 231-232 (Sarman, A., & Uzuntarla, Y. (2022). Attitudes of healthcare workers towards complementary and alternative medicine practices: A cross-sectional study in Turkey. European Journal of Integrative Medicine, 49, 102096. [https://doi.org/https://doi.org/10.1016/j.eujim.2021.102096](https://doi.org/https:/doi.org/10.1016/j.eujim.2021.102096). Source address: <https://www.sciencedirect.com/science/article/abs/pii/S1876382021008143>).

Response: we appreciate the comment and we have incorporated it in the revised copy.

In a study conducted in Turkey through a cross-sectional study, the knowledge levels of healthcare workers towards complementary and alternative medicine were adequate.
(8) The manuscript is presented in an intelligible fashion and written in standard English.
(9) I cannot advise on statistical analysis of the paper.

Reviewer #2: Comment #1
The title of the study should have to be changed in to “knowledge, Attitude and practice towards complementary and alternative medicine and associated factors among health care professionals in public health facilities of Hadiya zone, South Ethiopia” in stage of knowledge, Attitude and practice towards complementary and alternative medicine among health care professionals in public health facilities of Hadiya zone, South Ethiopia

Response: we appreciate the comment and we have incorporated it in the revised copy.

Comment #
In abstract the objective of the study should be stated in clear and unambiguous way and it has to be similar to the title of the study; in this paper the objective of the study were mixed with significance of the study; and the author should be amend accordingly as follows;
Even if modern medicine is becoming more widely available, a considerable portion of the world's population continues to rely on complementary and alternative medicine. Complementary and alternative medicine is used by 80% of the population in developing countries for their health care. The study was conducted to assess Knowledge, Attitude and practice towards complementary and alternative medicine among health care professionals in public health facilities of Hadiya zone, South Ethiopia.

Response: we appreciate the comment and we have incorporated it in the revised copy.

Comment #3
Methods
The methods part should be starting by stating the study area first and; the authors should have to be corrected as “The studies were conducted in Hadiya zone from October 10 to October 30, 2019. A facility-based cross-sectional study design was employed using simple random sampling methods. Three hundred sixty six health care professionals were selected using a simple random sample method. We used descriptive statistics, as well as bivariate and multivariate logistic regression analysis. An association was determined using P-values less than 0.05 and 95 percent confidence intervals. The results of the study were presented using texts, graphs and tables.

Response: we appreciate the comment and we have incorporated it in the revised copy.

Comment #4, in result part of abstract
Results
Three hundred and fifty-five respondents were interviewed, with a 96 percent response respondent rate. Two hundred thirty two (65.4%) of the study participants have good knowledge towards complementary and alternative medicine, 216 (60.8%) have a favorable attitude toward complementary and alternative medicine, and 182 (51.3%) have utilized it in the previous two years. Some of the factors associated with knowledge, attitude, and practice towards complementary and alternative medicine were health care professionals who have contact with patients who seek complementary and alternative medicine [AOR = 1.89(95% CI; 1.18, 3.03), female health care professionals [AOR = 2.43(95% CI; 2.68, 9.74), and more than six years work experience [AOR = 1.68(95% CI; 1.04, 2.71).

Response: we appreciate the comment and we have incorporated it in the revised copy.

Comment #5
In the background of the study in introduction part in first paragraph; in second sentence that said as a result, health46 care executives have been focusing on integration and looking for the positive elements of 47 CAM [1, 2, 3] in this way because it is already appear for the third times in the paragraph and this comment should be followed through ought the study.

Response: we appreciate the comment and we have incorporated it in the revised copy.

Comment #6
In the last paragraph in the last sentence of background the sentence which states the objective of the study that were mentioned by authors is better if it is changed as “the objective of this study were to assess health care workers knowledge, attitude, and practice of complementary and alternative medicine and associated factors among in public health facilities in Hadiya zone, south Ethiopia”.

Response: we appreciate the comment and we have incorporated it in the revised copy.
Comment #7
In methods and material of the study design and participants part the author’s should have re-write the idea that is mentioned in this subtitle because the study design and participants part should have to clearly described.

Response: An institution-based cross-sectional study design was used. All health care professionals who work in Hadiya zone public health facilities were considered as the source population, and sampled health care professionals in the randomly selected public health facilities were taken as the study population.

Comment #8
In Result part
Socio-demographic characteristics
A standardized questionnaire was used to interview rather than 350, it has to corrected as “355” respondents, resulting in a rather than 96 percent; has to be corrected as “97” percent response rate. Sixty-one percent of health-care providers should have to changed as “Two hundred thirty one (65.1%) had a bachelor's degree”. Two hundred twenty-two (62.5%) of the study participants have worked for six years 162 or more. Two hundred and twenty-eight (64.2%) of the participants in the study were male. Two 163 hundred thirty-two (65.4%) were married, and two hundred twenty-two (62.5%) were Hadiya by ethnic group, the sentence which describe about ethnicity should be deleted because it is not included in the table (Table 1).

Response: we appreciate the comment and we have incorporated it in the revised copy.
